# The Educational Treatment of Idiots, Imbeciles, and Feeble-Minded Children

**Published:** 1894-08-18

**Authors:** G. E. Shuttleworth

**Affiliations:** late Medical Superintendent, Royal Albert Asylum, Lancaster


### The Educational Treatment of Idiots, Imbeciles, and Feeble-Minded Children.

By G. E. Shuttleworth, B.A., M.D., &c., late Medical Superintendent, Royal Albert Asylum, Lancaster.

### IV.—THE INDUSTRIAL TRAINING OF THE IMBECILE.

We propose in the present paper to consider more particularly the industrial training of the imbecile and feeble-minded class, to which some reference has already been made in connection with their school instruction. To the low-grade idiot, indeed, industrial training, in its ordinary acceptation, is hardly applicable; yet there are many small matters, such as dusting furniture, picking up stones and sticks from grass land, &c., which require but little intelligence for the doing, and which when accomplished give a sense of satisfaction in respect of "something attempted, something done," that materially enhances the enjoyment of life. Moreover, it must be remembered that the adage,

Satan finds some mischief still
For idle hands to do, is specially true in the case of the idiot, and the child who is not provided with employment for his fingers will probably find it for himself in the form of tearing his clothing.

In idiot asylums, therefore, hair-teasing, or the picking of wool or silk for mattress-making, forms the lowest round in the handicraft ladder. Then comes such simple employment as shoe cleaning, and though often the hands and face of the operator absorb more blacking then the shoe operated on, it is encouraging to find that a fair proportion of imbecile boys so employed ultimately become qualified for the institution "Shoeblack Brigade." And it is interesting to see how semi-paralysed boys, pushing their paralysed hand into the boot, will vigorously ply their industry with the other. Next in order of difficulty come such occupations as sawing and chopping firewood, which, of course, imply more dexterity as well as some power of self-control. Plaiting coir for matmaking, basketwork, and sash-cord making are other industries successfully carried out by imbeciles, as also the making of brushes and brooms, the latter requiring, however, more than the average amount of nicety to ensure a workmanlike finish. Shoe-making, tailoring, and carpentry imply a still higher degree of intelligence; yet in the larger establishments all the shoes and clothing for the inmates are made and repaired on the premises, and the furniture is manufactured and kept in order, mainly by the work of the patients, supervised and assisted (it may be) by their instructors. Considerable skill is not infrequently shown in wood-carving, and it is astonishing to see the results attained in this art by lads originally the subject of spasmodic movements of the fingers, which we have referred to as "athetosis." Sometimes quite exceptional mechanical aptitude is displayed by imbeciles, and some of our readers who have visited Earlswood may be familiar with the wonderful models made by the so-called "Idiot Shipbuilder," whose self-taught notions of naval architecture justify the belief that with a well-balanced brain he might have taken a high position in the constructive branch of the Admiralty. This is by no means a unique instance of mechanical ability amongst the inmates of idiot asylums; and it has happened more than once that chief prizes in public industrial exhibitions have been awarded to imbecile competitors. It may be remarked in passing that where original ornamentation is attempted by imbeciles, there is a tendency to run into Oriental forms, Indian or Japanesque.

Interesting as indoor employment may be, there is no doubt that as a general rule the most beneficial, for mental as well as physical development, is occupation in the open air. We have already referred to the lowly labour of stone and stick gathering; then comes weeding, which at first requires smart supervision to prevent the plant disappearing with the weed. This difficulty surmounted, the grades of garden work with rake, hoe, fork, and spade follow naturally, and there are instances of institution-trained imbeciles following with pride and pleasure the profession of gardener. Farming also offers a large scope of industries adapted to the idiosyncracies of the imbecile, and while one lad will feed the stock with clock-work regularity, another will be "death on weeds" amongst the crops, and a third, of "horsey" tendencies, an efficient helper in the stables. In the depressed condition of agriculture in England, it would be rash to say that a farm colony, consisting of reclaimed imbeciles, would absolutely pay its way; but in America we find superintendents of "feeble-minded institutions" undertaking to defray the maintenance of working inmates out of the profits of land granted them by the State. In the Far West, indeed, the California Legislature has appropriated no less than 1,670 acres for its "Home for Feeble-minded Children," an estate naively described as having "everything on it that the Garden of Eden had, except perhaps the forbidden fruit!" At any rate, grapes, peaches, olives, not to speak of apples and pears, grow there galore; and the superintendent records that "splendid aid is given by the pupils in gardening, cutting, handling, and curing fruit," and that one year's output is estimated at about ten tons of dried fruit, and from three to four thousand gallons of canned goods and preserves.

The work of the girls must not be forgotten. Starting with such minor domestic matters as dusting and sweeping, the mysteries of bed making, house cleaning, and even laundry and kitchen work are gradually surmounted, and the effective labour of the female inmates of institutions materially diminishes the amount of paid service that would otherwise be required. Sewing and knitting, learnt in school, are also turned to practical account in connection with wardrobe necessities, and in many cases needlework of an extremely neat character is turned out by imbecile girls originally unable so much as to hold a needle with their rebellious fingers. The excellent character of the macramé work done by children of this type has been previously referred to; and recently, at the exhibition of work done in the London Board schools, the specimens contributed by the feeble-minded and defective children under instruction in the special classes deservedly attracted much notice and commendation. The stall which they occupied was, indeed, one of the most remarkable features of the show.

